# Tilt, Decentration, and Internal Higher-Order Aberrations of Sutured Posterior-Chamber Intraocular Lenses in Patients with Open Globe Injuries

**DOI:** 10.1155/2017/3517461

**Published:** 2017-10-29

**Authors:** Xiangjia Zhu, Yinglei Zhang, Wenwen He, Hongfei Ye, Chunhui Jiang, Yi Lu

**Affiliations:** ^1^Department of Ophthalmology, Eye, Ear, Nose, and Throat Hospital of Fudan University, 83 Fenyang Road, Shanghai 200031, China; ^2^Key Laboratory of Myopia, Ministry of Health, 83 Fenyang Road, Shanghai 200031, China; ^3^Shanghai Key Laboratory of Visual Impairment and Restoration of Shanghai, Fudan University, 83 Fenyang Road, Shanghai 200031, China

## Abstract

**Purpose:**

To evaluate the tilt, decentration, and internal higher-order aberrations (HOAs) of sutured posterior-chamber intraocular lenses (IOLs) in patients with open globe injuries.

**Methods:**

46 consecutive patients (47 eyes) who underwent transsclerally sutured IOL implantation were enrolled in this prospective cohort study. Nineteen eyes had a history of open globe injury. The tilt and decentration of the IOLs and the visual quality were measured 1 month after surgery.

**Results:**

The horizontal tilt and decentration of the IOLs in the open-globe-injury group were significantly higher than those in the control group (both *P* < 0.05). In the open-globe-injury group, the horizontal decentration was significantly greater in the limbus-sclera-involved group (*n* = 11) than in the only-cornea-involved group (*n* = 8, *P* = 0.040). The internal coma, 3rd-order, and total HOA values at pupil sizes of 4 mm (*P* = 0.006) and 6 mm (*P* = 0.013) were significantly higher in the open-globe-injury group than in the controls. Consequently, the optical quality data for the modulation transfer function and the Strehl ratio (all *P* < 0.05) were significantly poorer in the open-globe-injury group.

**Conclusions:**

Open globe injuries damage the structural integrity of the eyeball, resulting in more-misaligned sutured IOLs and poorer visual quality.

## 1. Introduction

In the absence of adequate capsular/zonular support, transsclerally sutured posterior chamber intraocular lens (IOL) implantation has become the universally preferred technique [1, 2]. However, IOL tilt and decentration tend to be more frequent and of greater magnitude after transscleral implantation than after normal in-the-bag implantation in response to the lack of capsular support [3, 4], and studies have shown that the tilt and decentration of the IOL can influence the optical quality of the eye and consequently cause unwanted optical images [5, 6].

Although absence of capsular/zonular support can result from various eye conditions, including Marfan syndrome [7], familial or idiopathic ectopia lentis, high myopia, vitrectomy surgery [8], and posterior capsule rupture during cataract surgery, one of the main causes is ocular trauma, which can be further classified into open and closed globe injuries [9]. Open globe injuries, such as ocular laceration or rupture, usually damage the structural integrity of the eyeball, a delicate optical system, which may affect the precise localization of a transsclerally sutured posterior-chamber IOL and cause poor optical quality of the eyeball [5].

Therefore, in this study, we evaluated the impact of open globe injuries on the tilt, decentration, and optical quality of eyes implanted with transsclerally sutured posterior-chamber IOLs.

## 2. Methods

This study was approved by the Institutional Review Board of the Eye and ENT Hospital of Fudan University, Shanghai, China, and was registered at ClinicalTrials.gov (accession number NCT02182921). All procedures adhered to the tenets of the Declaration of Helsinki. After being informed of the intention of the present study, each patient read and signed an informed consent.

### 2.1. Subjects

Forty-seven eyes of 46 consecutive patients that underwent uneventful implantation of transsclerally sutured posterior-chamber IOLs at the Eye and ENT Hospital of Fudan University between April 2013 and November 2014 were recruited. All the patients enrolled had a preoperative best-corrected visual acuity (BCVA) of ≥20/100. The eyes of patients with severe fundus pathologies, strabismus, glaucoma, or diabetes were excluded from the study. The selected eyes were divided into two groups according to the reason for the lack of capsular support: (1) the open-globe-injury group (IOL implantation was performed at least 3 months after lens removal), which consisted of 15 eyes with penetrating injuries and four with ocular ruptures, (ocular scars or small tissue defects did not pass through the IOL vortex and the pupillary axis) and (2) the control group, which consisted of 2 eyes with Marfan syndrome and 26 eyes after successful vitrectomy surgery for rhegmatogenous retinal detachment.

### 2.2. Surgical Technique

The implantation of the IOL was performed according to Szurman et al. [10]. First, a transscleral suture through the bed of two scleral flaps (at 3:00 and 9:00) was performed with the classic ab externo technique. The loop of the suture was then externalized through a 2.6 mm temporal clear corneal incision, with a hook, and cut in the middle. A hydrophilic acrylic foldable IOL (Rayner, Rayner Intraocular Lenses Ltd., Hove, UK) was inserted into the cartridge with both haptics extended. The IOL was advanced inside the cartridge until the leading haptic end disclosed at the anterior orifice for the first suture tie. The IOL was implanted by introducing the cartridge through the incision, and the extended trailing haptic was retained inside the tunnel incision for the second suture tie. The two suture ties were just at the corner of the haptic orifice. The trailing haptic was then inserted into the eye and the IOL was fixed into the ciliary sulcus with centered optics. Finally, the polypropylene suture was tied inside the scleral bed 1.5 mm posterior to the limbus, at 3 and 9 o'clock, somewhere other than the primary site of the wound, and the knot was buried.

### 2.3. Postoperative Examinations

One month after surgery, all patients underwent an ophthalmological examination including uncorrected visual acuity (UCVA), BCVA, intraocular pressure (IOP), slit-lamp biomicroscopy, and manifest refraction. Snellen visual acuity measurements were converted to logarithm of the minimum angles of resolution (logMAR) equivalents for data analysis. Anterior segment tomography (Pentacam HR, Oculus, Wetzlar, Germany) and wavefront aberrometry (KR-1W, Topcon, Tokyo, Japan) were performed before and after mydriasis.

### 2.4. Tilt and Decentration Measurements

The Scheimpflug imaging system was used to image the anterior segment of the eye at different meridians [11]. During the measurements, two Scheimpflug slit-lamp images at slit angles of 90° and 180° were obtained and evaluated with the Image-Pro Plus software (Media Cybernetics Inc., Rockville, MD, USA). After geometric correction, the contours of the original images were enhanced further with binarization and curve-fitting techniques. The anterior and posterior surfaces of the IOL were plotted to determine its optical axis. The pupillary axis was calculated as the line passing through the anterior corneal center of curvature and the center of the pupil. The tilt of the IOL optical axis relative to the pupillary axis and the distance between the IOL vortex and the pupillary axis were calculated.

### 2.5. Evaluation of Visual Function

Visual quality was assessed with a KR-1W aberrometer, which provided both modulation transfer function (MTF) curves and Strehl ratio values, obtained with the point spread function (PSF), as indices of the optical quality of the eyes. Internal higher-order aberrations (HOAs) were calculated for pupil diameters of 4.0 mm and 6.0 mm using the Zernike coefficients from the 3rd to the 4th order. The root-mean squares of the 3rd-order Zernike components, (Z3^−1^ to Z3^1^) and (Z3^−3^ to Z3^3^), were used to represent the coma-like and trefoil-like aberrations, respectively. The root-mean squares of the 4th-order Zernike components, (Z4^0^) and (Z4^−4^ to Z4^4^), were used to represent the spherical-like and tetrafoil-like aberrations, respectively.

### 2.6. Statistical Analysis

All analyses were performed with SPSS version 11.0 (SPSS, Chicago, IL, USA). Categorical values were interpreted as proportions, whereas continuous variables were expressed as the means ± standard deviations (SD). Student's *t*-test was used to compare variables between the two groups. A paired *t*-test was used to compare the preoperative and postoperative variables. A *P* value of 0.05 or less was considered statistically significant.

## 3. Results

### 3.1. Baseline Characteristics

The mean age of the enrolled patients at surgery was 51.7 ± 12.1 years. Of the 46 patients evaluated, 37 (80.4%) were male and 19 (41.3%) had open globe injuries. Compared with the preoperative variables, the postoperative corneal endothelial cell density (ECD) was significantly reduced (*P* < 0.001, paired *t*-test), but the postoperative IOP (*P* = 0.095, paired *t*-test) and BCVA (*P* = 0.915, paired *t*-test) were not significantly altered (Table 1).

### 3.2. Tilt and Decentration of IOL

**Figure 1:**
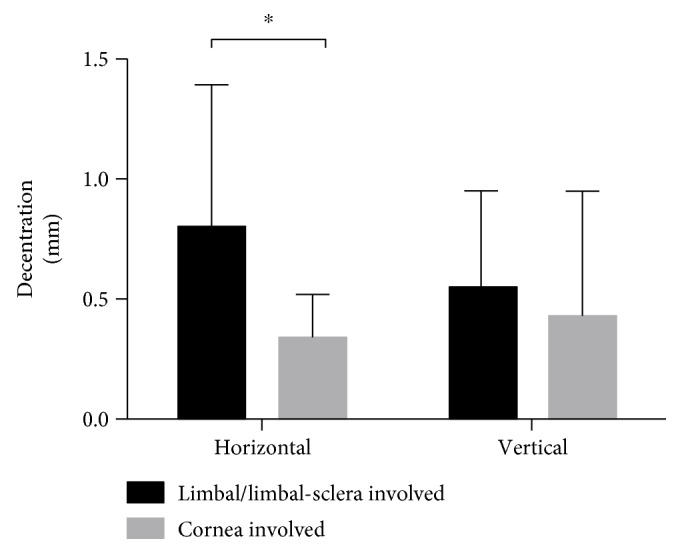
Horizontal and vertical decentration of IOLs in the limbus/limbus-sclera-involved group and the only-cornea-involved group. Horizontal decentration was significantly higher in the limbus/limbus-sclera-involved group than in the cornea-involved group (*P* = 0.040). ^∗^*P* < 0.05.

Table 2 shows the horizontal and vertical tilt and decentration in the open-globe-injury group and control group. In the 46 patients, the average horizontal tilt angle was 1.73 ± 1.54° and the decentration length was 0.47 ± 0.40 mm, and the average vertical tilt was 2.33 ± 2.10° and decentration length was 0.39 ± 0.45 mm. Statistically significant differences between the open-globe-injury group and the controls were only observed in the horizontal tilt (*P* = 0.041, Student's *t*-test) and decentration (*P* = 0.029, Student's *t*-test). When we compared the tilt and decentration between the limbus/limbus-sclera-involved group (*n* = 11) and the only-cornea-involved group (*n* = 8), the horizontal decentration was significantly greater in the limbus/limbus-sclera-involved group (*P* = 0.040, Student's *t*-test) (Figure 1). The horizontal and vertical tilt did not differ significantly between the two groups (both *P* > 0.05, Student's *t*-test).

### 3.3. Internal HOAs

**Figure 2:**
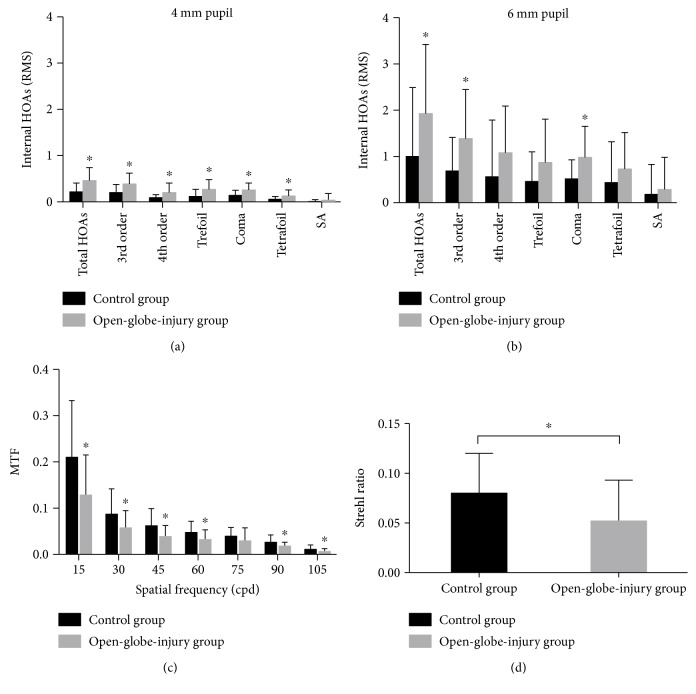
Internal higher-order aberrations (HOAs) and optical quality in the control and open-globe-injury groups. (a) and (b) show the internal HOAs in the two groups at pupil diameters of 4 mm (a) and 6 mm (b). At a premydriatic pupil size of 4 mm, most internal HOA values were significantly higher in the open-globe-injury group than in the controls. After mydriasis, only total HOAs, third-order HOAs, and coma differed significantly between the two groups. In (c), MTF at spatial frequencies of 15, 30, 45, 60, 90, and 105 cycles/degree were significantly lower in the open-globe-injury group than in the controls. (d) indicates significantly lower Strehl ratio in the open-globe-injury group than in the controls. ^∗^*P* < 0.05.

We also compared the internal HOAs between the open-globe-injury group and the control group. Statistically significant differences were found in all internal HOA items, except the spherical aberrations (*P* = 0.262), between the two groups at a premydriatic pupil size of 4 mm (*P*_total HOAs_ = 0.001, *P*_3rd order_ = 0.003, *P*_4th order_ = 0.024, *P*_trefoil_ = 0.009, *P*_coma_ = 0.006, *P*_tetrafoil_ = 0.033, Student's *t*-test; Figure 2(a)). After mydriasis, statistically significant differences between the two groups at a pupil diameter of 6 mm were only found in the coma, third-order, and total HOAs (*P*_coma_ = 0.013, *P*_3rd order_ = 0.019, *P*_total HOAs_ = 0.046, Student's *t*-test; Figure 2(b)).  Figure 2(c) shows the comparison of MTF in the two groups and indicates significant differences at spatial frequencies of 15, 30, 45, 60, 90, and 105 cycles/degree (*P* = 0.009, *P* = 0.049, *P* = 0.020, *P* = 0.032, *P* = 0.030, and *P* = 0.049, resp., Student's *t*-test). The PSF data were also poorer in the open-globe-injury group (0.052 ± 0.041) than in the controls (0.080 ± 0.040, *P* = 0.033, Student's *t*-test; Figure 2(d)).

**Figure 3:**
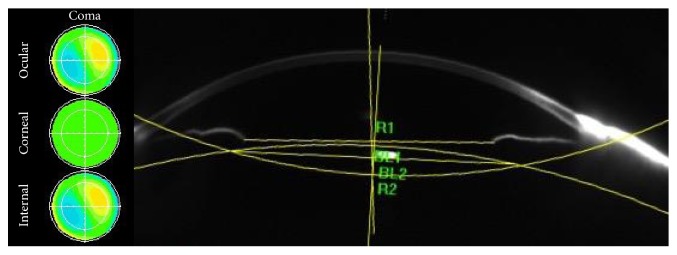
Illustration of the coma aberrations and measurements of tilt and decentration in a Scheimpflug image of a typical patient from the open-globe-injury group. Horizontal tilt was 2.85° and horizontal decentration was 0.50 mm; vertical tilt was 1.5° and vertical decentration was 0.47 mm. Internal coma is a major source of ocular coma.

The coma values, tilt, and decentration in the right eye of a patient with open globe injury are illustrated in Figure 3. A horizontal tilt angle of 2.85° and a horizontal decentration length of 0.50 mm and a vertical tilt angle of 1.5° and a vertical decentration length of 0.47 mm were determined from the Scheimpflug images. Ocular coma mainly stemmed from the internal coma, which was closely related to the tilt of the IOL.

## 4. Discussion

In the absence of adequate capsular/zonular support, a transsclerally sutured posterior-chamber IOL usually produces greater tilt and decentration than a normal in-the-bag implanted IOL [12–14]. Consequently, the optical quality in the former is usually poorer than that of the latter. One of the main causes of insufficient capsular support is ocular trauma, especially open globe injuries, which can damage the structural integrity of the eyeball, an otherwise perfect optical system, and consequently influence the localization of transsclerally sutured posterior chamber IOLs [15, 16]. However, few studies have investigated the effects of open globe injury on the tilt and decentration of transsclerally sutured IOLs or its influence on the optical quality of the eyeball. In the present study, we identified significantly poorer positioning and on-axis alignment of transsclerally sutured IOLs in patients with open globe injuries than in those without, which induced higher internal coma and worse visual quality in these eyes.

“Open globe injury” refers to a full-layer opening of the eyeball. The lens is easily damaged during the injury or is removed later in the treatment of complications arising from the primary injury. Although in most cases the eyeball roughly recovers its structural integrity, either with surgical treatment by an eye specialist or with self-sealing in small wounds, the delicate optical structure of the eyeball as a whole is usually damaged by scars or small tissue defects, so it is no longer a perfect optical system [15, 17]. This is the usual precondition in most eyeballs with a history of open globe injury undergoing the implantation of transsclerally sutured IOLs, and the functional prognosis is poorer for them than for untraumatized eyes. Because the IOL is fixed transsclerally, adjacent to the limbus, open globe injuries involving the limbus are most likely to affect the precise localization of the IOL.

Tilt and decentration are important predictors of the precise localization of an IOL. Transsclerally sutured IOLs normally show a significant amount of tilt and decentration in the absence of capsular support [18–20]. Hayashi et al. [21] reported an average tilt angle of 6.35° and decentration length of 0.62 mm in eyes with sclerally sutured IOLs, which were significantly higher than those of in-the-bag IOLs, which had a tilt angle of 3.18° and decentration length of 0.29 mm. Durak et al. [12] evaluated the tilt and decentration after the primary and secondary implantation of transscleral IOLs. The tilt and decentration were 5.71 ± 3.41° and 0.59 ± 0.38 mm, respectively, in the primary implantation group and 6.22 ± 3.94° and 0.69 ± 0.45 mm, respectively, in the secondary implantation group. In the present study, the average horizontal tilt angle was 1.73° and the decentration length was 0.47 mm, which compare favorably with those in previous studies.

The tilt and decentration were significantly greater in the open-globe-injury group than in the control group, and within the open-globe-injury group, the tilt and decentration of the IOLs in the limbus/limbus-sclera-involved cases were even greater than those in the only-cornea-involved cases. Because the IOL was sutured about 1.5 mm posterior to the limbus, any trauma involving that region could compromise the position of the IOL. Although the site of suture during surgery was selected somewhere other than the primary site of the wound, the tilt and decentration of the IOLs were still high in the open-globe-injury group, suggesting that the influence of the wound extended beyond its location. Therefore, the influence of the ocular integrity and wound position on the fine localization of transsclerally sutured IOLs was further highlighted. In contrast, the IOL was usually sutured at 3 and 9 o'clock, which might explain the finding that only the horizontal tilt and decentration were higher in the traumatized group. Geometrically, two points only define a line, whereas three points define a plane. To avoid such tilt and decentration in the future, an IOL with three or more loops, which would allow three sutures or an intrasclerally fixed IOL, might be considered.

Previous studies have shown that certain visual complaints are associated with corresponding HOAs with specific Zernike coefficients (e.g., monocular diplopia is associated with coma [22, 23]) and that IOL misalignment can cause visual impairment and an obvious level of coma-like aberration [6, 24–26]. Because the transsclerally sutured IOL is located within the eyeball, internal HOAs might be appropriate indicators of the influence of tilt and decentration on the optical quality, regardless of the impact of the damaged eyeball wall. Consistent with the data for tilt, we found that the internal coma aberration was significantly greater in the open-globe-injury group than in the control group. Consequently, the internal 3rd-order and total HOAs were also higher in the open-globe-injury group with pupil diameters of both 4 and 6 mm. Based on these findings, it would be interesting to determine the influence of IOL misalignment and coma aberration on the visual quality of patients after the implantation of transscleral sutured IOLs. As indicated by the wavefront sensor, the MTF curve and PSF data were also poorer in the open-globe-injury group, which suggests that eyes with a perfect undamaged natural contour will display better optical performance after the scleral suture fixation of IOLs. Because this was a single-center prospective study with a limited number of subjects and only one type of foldable lens was used, our results should be verified by others.

In summary, transsclerally sutured IOL implantation in eyes with a history of open globe injury might show poorer IOL positioning and higher internal coma than those with no such history, leading to worse visual quality after surgery than in uninjured eyes.

## Figures and Tables

**Table 1:** Clinical outcomes.

Parameters	Preoperative	Postoperative	*P* values
BCVA (logMAR)	0.37 ± 0.32	0.36 ± 0.36	0.915
IOP (mmHg)	15.79 ± 4.70	17.78 ± 6.81	0.095
ECD (cells/mm^2^)	2384.15 ± 331.44	2244.41 ± 399.55	<0.001
SE (D)	(+)11.00 ± 3.28	(−)1.22 ± 1.21	—

BCVA: best-corrected visual acuity; logMAR: logarithm of the minimum angle of resolution; IOP: intraocular pressure; ECD: endothelial cell density; SE: spherical equivalent.

**Table 2:** Tilt and decentration 1 month after IOL implantation.

Parameters	Etiology classification		*P* value
	Controls	Open globe injury	
Horizontal tilt (°)	1.26 ± 1.06 (0.37–4.22)	2.46 ± 1.93(0.50–6.84)	0.041^∗^
Horizontal decentration (mm)	0.35 ± 0.27 (0.01–0.96)	0.67 ± 0.49 (0.17–1.70)	0.029^∗^
Vertical tilt (°)	2.40 ± 2.36 (0.05–7.92)	2.30 ± 1.77 (0.24–5.39)	0.89
Vertical decentration (mm)	0.34 ± 0.44 (0.02–2.09)	0.50 ± 0.46 (0.09–1.64)	0.29

Tilt and decentration of IOLs were compared between the two groups with Student's *t*-test. ^∗^*P* < 0.05.
